# Thermodynamic and crystallographic model for anion uptake by hydrated calcium aluminate (AFm): an example of molybdenum

**DOI:** 10.1038/s41598-018-26211-z

**Published:** 2018-05-21

**Authors:** Nicolas C. M. Marty, Sylvain Grangeon, Erik Elkaïm, Christophe Tournassat, Clémence Fauchet, Francis Claret

**Affiliations:** 1 BRGM, 3 Avenue Guillemin, Orléans, Cedex 2 45060 France; 2 Synchrotron SOLEIL, L’Orme des Merisiers Saint-Aubin, BP 48, 91192 Gif-sur-Yvette Cedex, France; 3 ISTO UMR 7327 Université d’Orléans–CNRS– BRGM, 45071 Orléans, France; 4 Lawrence Berkeley National Laboratory, 1 Cyclotron Road, Mail Stop 90-1116, Berkeley, CA 94720 United States

## Abstract

Amongst all cement phases, hydrated calcium aluminate (AFm) plays a major role in the retention of anionic species. Molybdenum (Mo), whose ^93^Mo isotope is considered a major steel activation product, will be released mainly under the form of MoO_4_^2−^ in a radioactive waste repository. Understanding its fate is of primary importance in a safety analysis of such disposal. This necessitates models that can both predict quantitatively the sorption of Mo by AFm and determine the nature of the sorption process (i.e., reversible adsorption or incorporation). This study investigated the Cl^−^/MoO_4_^2−^ exchange processes occurring in an AFm initially containing interlayer Cl in alkaline conditions using flow-through experiments. The evolution of the solid phase was characterized using an electron probe microanalyzer and synchrotron high-energy X-ray scattering. All data, together with their quantitative modeling, coherently indicated that Mo replaced Cl in the AFm interlayer. The structure of the interlayer is described with unprecedented atomic-scale detail based on a combination of real- and reciprocal-space analyses of total X-ray scattering data. In addition, modeling of several independent chemical experiments elucidated that Cl^−^/OH^−^ exchange processes occur together with Cl^−^/MoO_4_^2−^ exchange. This competitive effect must be considered when determining the Cl^−^/MoO_4_^2−^ selectivity constant.

## Introduction

With more than seven billion cubic meters produced annually, cement is probably the most widely used material on Earth^1^. Cement-based materials are ubiquitous in construction, including in the design of access structures, galleries, vaults, and waste packages of deep underground radioactive waste disposal sites. In this context, cement-based materials are chosen primarily for their mechanical resistance. However, additional properties of interest are their low permeability, together with their strong chemical reactivity manifest via their cation and anion sorption properties^2–21^, which contribute to the concept of a multiple barrier system between the waste matrix and the biosphere^22^.

Amongst all cement phases, hydrated calcium aluminate (AFm) plays a major role in the retention of (radioactive) anions that enter into contact with cement-based materials. AFm is member of the layered double hydroxides (LDHs) group, meaning that its structure consists of stacked layers of positively charged atoms separated from each other by anion-containing hydrated interlayer spaces. The general structural formula of an AFm is [Ca^2+^_4_(Al^3+^_x_Fe^3+^_(1−x)_)_2_(OH)_12_]∙A∙*n*H_2_O, where the layered species are between the brackets and A∙*n*H_2_O represents the hydrated exchangeable “interlayer anions.” These exchangeable interlayer anions compensate for the layer charge induced by the presence of the trivalent cations in the layers^8,9,17–21,23–25^, providing AFm with an anion-exchange capacity (AEC). If monovalent, the stoichiometry of the interlayer anions is equal to that of trivalent layer cations.

Of all AFm, the most studied is probably the one with the general structural formula [Ca^2+^_4_Al^3+^_2_(OH)_12_]∙2Cl^−^∙*n*H_2_O. This phase (hereafter, AFm-Cl) has been given various names depending on the layer stacking mode and lattice parameters, degree of hydration, and its natural or synthetic occurrence. Synthetic samples are often termed AFm-Cl or “Friedel’s salt,” and natural samples are normally said to belong to the “hydrocalumite” group. A detailed description of the nomenclature pertaining to this type of mineral is available elsewhere^26^. While natural occurrences of hydrocalumite are scarce, synthetic forms are abundant in cement-based materials where they control the retention of anions such as iodine^15,27^, molybdenum (Mo)^21,28^, selenate^29^, and arsenic^30,31^.

Understanding, and thus being able to model the mechanisms controlling these retention properties, requires a sound description of the sorption process from crystallographic to macroscopic (aqueous geochemistry) scales. This methodological approach, which has been used successfully to study the mechanisms of ion adsorption by clay minerals^32–36^, iron and manganese (hydr)oxides^37–40^, and Mg-Al LDH^41^, has to date been seldom applied to cement phases. One noticeable exception is the case of Ca uptake by C-S-H (the main cement component), for which the geochemical and crystallographic studies, although conducted separately^42–46^, could be combined to propose a coherent model. The molecular-scale crystallographic descriptions in these studies allow the exact retention mechanism (reversible sorption – i.e., adsorption, or incorporation in the lattice, or recrystallization) to be determined and, in case of adsorption, provide the structural conformation of the interlayer anions. In turn, the selectivity constants determined from the chemistry are required to predict the partitioning of the element of interest between the solid and liquid phases, provided the crystallographic analysis could determine that the element is in an exchangeable position. In the case of AFm, the coupled crystallographic and geochemical approach has, to the best of our knowledge, been applied solely to the determination of the solubility constants of AFm in which the interlayer Cl was exchanged with various anions, including iodine^8,14,15,27^, Mo^21^, and selenite^47^. Thus, there have been no studies linking the determination of anion sorption sites, macroscopic geochemical selectivity constants, and the number and density of sorption sites involved in the retention mechanisms. However, such information is required to build robust predictive models for the binding and release of anions to AFm-Cl and, more generally, to cement-based materials.

Among all the anions of concern regarding (radioactive) waste storage, Mo is of special interest: ^99^Mo is used widely as a precursor of ^99m^Tc and ^93^Mo is an activation product of spent nuclear fuel^21^. In near-neutral to basic conditions, as might be expected in storage sites and in cement pore water, MoO_4_^2−^ is the main Mo species, and it could be retained by anion-sorbing phases or by precipitation under the form AFm-MoO_4_ and/or powellite (CaMoO_4_) at highest concentrations^21^. Determining its mechanisms of interaction with AFm-Cl, and thus with cement-based materials, is consequently of prime importance. In this regard, Ma *et al*.^21^ undertook a number of experiments that consisted of contacting AFm-Cl with MoO_4_^2−^ in closed systems (laboratory batch experiments and *in situ* synchrotron experiments using closed reactors). At Mo concentrations in the range 0.003–10 mM, they observed AFm dissolution/reprecipitation phenomena, as well precipitation of a secondary phase (i.e., powellite). This prevented detailed analysis of the exchange reactions.

To improve the understanding of Mo uptake by AFm-Cl via exchange mechanisms, this study performed Cl^−^/MoO_4_^2−^ exchange experiments using a stepwise and multidisciplinary approach, i.e., an AFm-Cl powder sample was introduced into a flow-through reactor, in which a solution of MoO_4_^2−^ was circulated, and the outlet-solution chemistries were monitored over time. Experiments were performed using medium Mo concentrations (0.6–0.9 mM) under far-from-equilibrium conditions with respect to AFm. Examination and modeling of the chemistry of the output solution, together with detailed characterization of the initial and final solids using laboratory and synchrotron techniques, allowed the mechanisms of MoO_4_^2−^ adsorption by AFm-Cl to be deciphered and the selectivity constants to be determined.

## Experimental Setup

### Flow-through experiments

**Figure 1:**
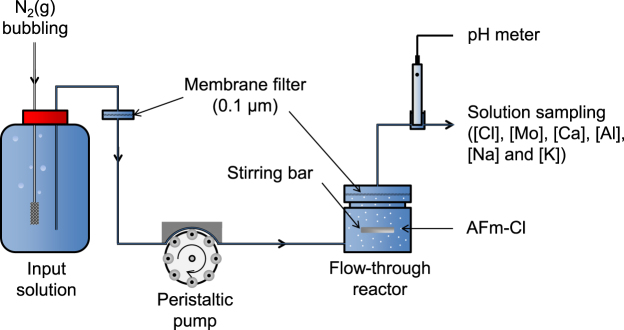
Experimental apparatus. Flow rates, pH, and Cl, Mo, Ca, Al, Na, and K concentrations were monitored as a function of time.

Exchange experiments were performed on AFm-Cl using flow-through reactors at room temperature (Fig. 1; also describe elsewere^48,49^). The total volume of the reactor was about 84 mL. Input solutions containing Mo were bubbled continuously under an N_2_ flux to avoid dissolution of the AFm and precipitation of calcite during the experiments (e.g.^50^) and to avoid the presence of CO_3_^2−^ in the interlayer^51^. Inlet solutions were injected through the reactors using a peristaltic pump (Watson Marlow, 205U) at a constant flow rate of about 2 mL min^−1^. AFm-Cl particles were maintained in suspension using a magnetic stirrer rotated on an axle to prevent any grinding of the material between the bar and the bottom of the reactor.

The initial masses, flow rates, and experimental durations are presented in Table 1.

**Table 1:** Experimental conditions of flow-through experiments.

Exp. n°	Initial Mass (g)	Flow rates* (mL min^−1^)	Duration (h)
**1**	0.142	2.03–2.01	6.9
**2**	0.242	2.00–1.96	6.9
**3**	0.499	2.23–2.16	6.9

*Monitored maximum and minimum flow rates.

As schematized in Fig. 1, the outlet solutions were filtered through a 0.1-μm membrane before being collected. The fluid sampling allowed the monitoring of solution chemistries (Cl, Mo, Al, Ca, Al, Na, and K concentrations) and flow rates as a function of time. All monitored data are reported in the Supplementary Information (Tables S1–S3).

On completion of the experiment, the solid suspension was collected and it was then filtered using a 0.1-µm filter. Subsequently, the solid samples were freeze-dried and then stored in an N_2_-filled glove box, which was maintained at a relative humidity of c.a. 10% using a saturated LiCl solution.

### Materials

#### AFm-Cl sample

AFm-Cl was synthetized following a previously described protocol^48^, which involves mixing stoichiometric amounts of tricalcium aluminate (C_3_A) and CaCl_2_·2H_2_O (1:1 molar ratio^37^) in water and at room temperature. All syntheses were performed in an N_2_-filled glove box using ultrapure water (resistivity = 18.2 MΩ cm), which was degassed prior to its introduction into the glove box. After 15 d maturation, the synthesized AFm-Cl was filtered, freeze-dried, and stored in an N_2_-filled glove box.

Because the synthesis of AFm-Cl involved the use of CaCl_2_·2H_2_O, and because the retrieved material was not washed after filtration, a weak presence of calcium chloride salt in the dried AFm could have been suspected. This was tested for by performing leaching experiments (Table 2). No linearity between the measured Cl and Ca concentrations as a function of the solid/liquid ratio was observed (S/L in g g^−1^ of water); thus, the presence of salts in the dried product was discounted.

**Table 2:** Experimental conditions and results of the leaching experiment.

Sample	Mass of AFm-Cl (g)	Mass of water (g)	Solid/liquid ratio	pH	Cl (mM)	Ca (mM)	Al (mM)	Ca/Cl	SI_AFm-Cl_*	SI_Gibbsite_*
Blank	0	10	0	n.d.	0	0	0	—	—	—
0.1	0.114	10	0.01	11.69	9.57	11.2	4.18	1.17	−0.50	0.99
0.2	0.160	10	0.02	11.73	10.9	11.2	3.76	1.03	−0.35	0.90
0.5	0.405	10	0.04	11.64	14.9	12.0	3.01	0.81	−0.52	0.89
1.0	0.985	10	0.10	11.61	22.8	14.7	1.63	0.64	−0.58	0.65

*Saturation indices (SI = log IAP/K, where IAP is the ionic activity product and K is the solubility constant).

n.d.: not determined, as the pH measurements using a classical glass electrode on high purity solutions are inappropriate^70^.

#### Reacting solutions

Two types of solution were prepared for each flow-through experiment: (i) an “input solution” enriched in Mo, which was injected inside the reactor, and (ii) an “initial reactor solution”, which initially filled the reactor. The solutions were prepared using MoO_3_, Ca(OH)_2_, NaCl, KCl, Al_2_O_3_, and ultrapure water. The fluids were bubbled with N_2_ for about 20 h before the experiments.

The chemistry of the reacting solutions is presented in Table 3. Note that NaCl and KCl were used as tracers to constrain the modeling of the flow-through experiments. Relative uncertainties were estimated at 10% from the discrepancies between the measured Cl and K/Na concentrations (Table 3).

**Table 3:** Solution compositions of reacting solutions.

Exp. n°	Solution type	Measured pH	Calculated pH*	Cl (mM)	Mo (mM)	Ca (mM)	Al (µM)	Na (mM)	K (mM)
**1**	Input solution	12.23	12.33	4.81	0.91	14.1	21.2	5.25	0
	Initial reactor solution	12.29	12.31	5.03	0	13.6	17.8	0	5.45
**2**	Input solution	12.08	12.30	5.03	0.59	13.2	10.8	5.23	0
	Initial reactor solution	12.12	12.30	5.63	0	13.2	7.75	0	5.45
**3**	Input solution	12.29	12.28	4.74	0.74	12.4	9.75	5.02	0
	Initial reactor solution	12.26	12.29	4.66	0	12.7	22.2	0	5.00

*PHREEQC calculation considering addition of Ca(OH)_2_ with respect to measured Ca concentrations.

AFm precipitation was not expected from the reacting solutions because AFm-Cl, AFm-OH, and AFm-MoO_4_ were undersaturated.

## Results and Discussion

To constrain the mechanisms of Mo incorporation into the AFm with initially interlayer Cl, and thus to allow successful interpretation of the chemical data, the solids collected on completion of all flow-through experiments, as well as an aliquot of unreacted sample, were analyzed for their mineralogy to elucidate the changes that occurred during the flow-through experiments.

### Mineralogical transformations

#### Chemical composition of initial and reacted samples

The unreacted AFm had a stoichiometric Ca to Al ratio of 2, as expected for defect-free AFm (Table 4). Coherently, the Cl to Al ratio was, within the limit of uncertainties, 1. This suggests the only source of layer charge was Al. After the flow-through experiments, in comparison with the unreacted material, the samples were depleted in Cl and enriched in Mo. Based on the Cl to Mo ratio, the degree of Mo for Cl exchange increased in the order of experiment 1 to 3 to 2 (Table 4).

**Table 4:** Average initial and final AFm compositions (results reported in mole of element per mole of AFm).

Exp. n°	Ca	Al	Cl	Mo	OH**
AFm-Cl (initial)*	4	1.8 ± 0.1	1.8 ± 0.2	0	0
1	4	1.9 ± 0.1	0.8 ± 0.5	0.4 ± 0.3	0.3
2	4	1.8 ± 0.1	0.2 ± 0.0	0.7 ± 0.1	0.2
3	4	1.9 ± 0.1	0.4 ± 0.1	0.6 ± 0.1	0.3

Note: data normalized such that Ca = 4 referring to the structural formula of AFm-Cl (Ca_4_Al_2_Cl_2_O_6_·10H_2_O).

*Data extracted from Marty *et al*.^48^: sample analyzed by EPMA was named AFm-Cl (b) by the authors.

**Calculation: OH = Al-Cl-2Mo.

Although the ratio of Cl removed to Mo incorporated was close to 2, suggesting that MoO_4_^2−^ replaced Cl^−^ as the interlayer charge-compensating anion, a deficit of anions in the interlayer position was suspected because all samples verified, on average, the following relation:

1 $$\sum _{i=1}^{n}{n}_{i}{z}_{i} < {n}_{Al}$$

where *n*_*i*_ is the number of moles of an anion *i* (Cl^−^ or MoO_4_^2−^) per mole of AFm, *z*_*i*_ is the charge of that ion (1 for Cl^−^, 2 for MoO_4_^2−^), and *n*_*Al*_ is the number of moles of Al per mole of AFm. As the Ca to Al ratio in the solids collected on completion of all the experiments remained similar to that of the unreacted sample, a change in the density of the layer charge was excluded. Another anion that cannot be probed straightforwardly by an electron probe microanalyzer (EPMA) was thus incorporated in the AFm during the experiments. This anion was OH^−^, because it is unquantifiable by EPMA (i.e., the loss of weight is unquantifiable because the AFms are highly hydrated) and because it is present in large concentrations in the input solutions used for the flow-through experiments (i.e., pH > 12). Moreover, the opposite exchange process (i.e., Cl^−^ sorption onto AFm-OH) has been reported by Suryavanshi *et al*.^52^.

#### Crystal structure

**Figure 2:**
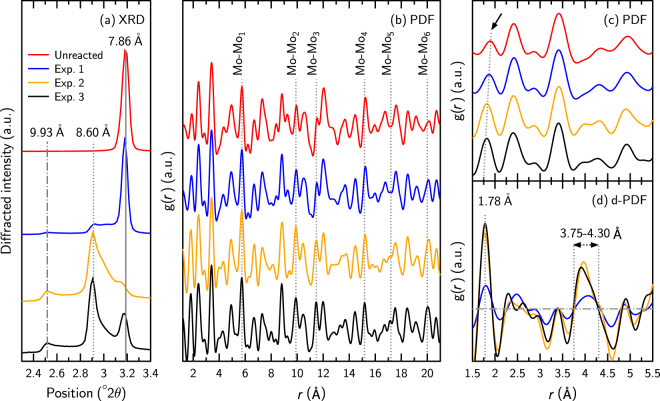
High-energy X-ray scattering data. In all panels, the red, blue, orange, and black solid lines (sorted from top to bottom in **a**,**b** and **c**) were collected respectively on the unreacted sample and on samples after experiments 1, 2, and 3. (**a**) Detail of the 2.3–3.4° 2θ region of the data, where three peaks attributable to three different layer-to-layer distances of 7.86 Å (solid gray line), 8.60 Å (dotted gray line), and 9.93 Å. (dash–dotted gray line) were identified. (**b**) PDF data of the same samples. The vertical dotted gray line reveals the positions of (from left to right) the first to sixth Mo-Mo correlations (see text for details). (**c**) Detail of the 1.5–5.5 Å region of the PDF data. The arrow identifies the main change that occurred in this low-r region: the strengthening and shift toward low r values of the correlation that was originally attributed to the shortest Al-O correlation, highlighted by the dotted gray line. (**d**) d-PDF data obtained by subtracting the data obtained on the fresh sample from the data obtained on the samples that underwent contact with Mo. Two correlations are visible in these d-PDF data: at 1.78 Å (dotted gray line) and in the 3.75–4.30 Å region (arrowed). The dash–dotted horizontal gray line represents the equation y = 0.

The X-ray diffraction (XRD) pattern of the unreacted material was typical of AFm-Cl, with the presence of an intense maximum at 3.19° 2θ (7.86 Å; Fig. 2a), assigned to the basal 002 reflection^53^. Upon exchange with Mo, the low angle region of the diffraction pattern underwent significant change (Fig. 2a). In the XRD pattern of the sample with the lowest Mo content (experiment 1), the 002 reflection of AFm-Cl was present, as were two new reflections at 2.52° 2θ and 2.91° 2θ (9.93 Å and 8.60 Å). These two new layer-to-layer distances were close to those of ~10.3 Å and ~9.1 Å observed previously during Mo incorporation into AFm-Cl^21^. The fact that the presently observed layer-to-layer distances were 5–6% smaller was certainly because the previous data were obtained on samples in suspension, which consequently incorporated more H_2_O in the interlayer^23^. The swelling from 7.86 Å to 8.60–9.93 Å was translated as a replacement of Cl^−^ by MoO_4_^2−^.

**Figure 3:**
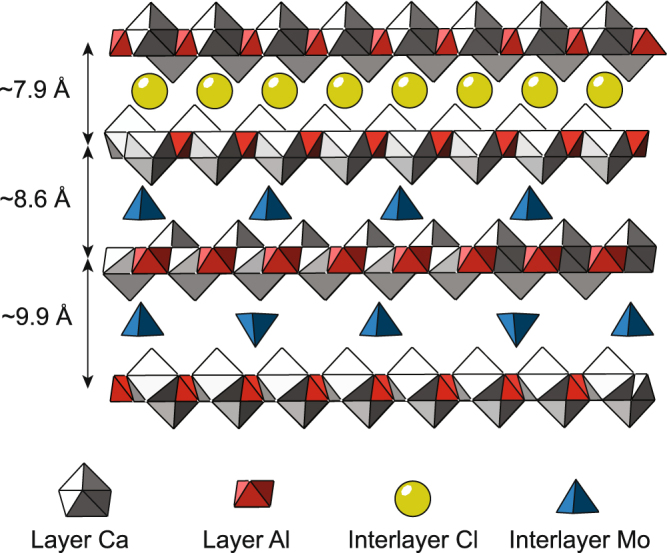
Proposed evolution of layer-to-layer distance as a function of interlayer anion nature and composition. Gray and red polyhedra respectively represent layered Ca and Al coordination spheres, while green spheres and blue tetrahedra reflect interlayer Cl^−^ and interlayer MoO_4_^2−^, respectively.

Two different layer-to-layer distances were observed for the Mo-exchanged structure (hereafter, referred to as AFm-Mo), indicating the presence of two different orderings of the MoO_4_^2−^ tetrahedra in the interlayer. For a Mo-O distance of 1.78 Å for the interlayer Mo in the tetrahedral coordination (see below), the height of an interlayer MoO_4_^2−^ polyhedron is 2.37 Å. The increase in the layer-to-layer distance from 8.60 Å to 9.93 Å possibly reflects the ordering of MoO_4_^2−^ polyhedra, with part of the polyhedra pointing toward a given layer and the other part pointing toward the opposite layer. In this assumption, the interlayer mid-plane passes through the middle of each tetrahedron (Fig. 3).

The expected layer-to-layer distance for an AFm containing interlayer OH^−^ is 7.9 Å^24^, i.e., identical to that of AFm-Cl. Consequently, the analysis of the XRD patterns could not provide information on the presence of OH^−^ in the interlayer.

Using the intensities of the peaks at 3.19° 2θ (7.86 Å) and 2.91° 2θ (8.60 Å) as indicators of the abundances of the interlayer Cl^−^ and MoO_4_^2−^, respectively, the degree of Cl/Mo exchange increased in the order: unreacted sample, experiment 1, experiment 3, and experiment 2, which also agreed with the EPMA data (Table 4). Note that the presence of synthetic powellite (CaMoO_4_) was not detected here, although it was detected in a previous study^21^ of a closed system, at Mo concentration over 1 mM. The lower Mo concentrations used here (i.e., <1 mM), together with the continuous renewing of reacting solutions that limited the increase of Ca concentration, prevented powellite precipitation.

All peaks attributable to 00 *l* reflections of AFm-Mo were asymmetric (Fig. 2a) and the peak at 7.86 Å was shifted towards low angles, which is indicative of interstratified structure. Given the high degree of asymmetry, the Reichweite parameter, describing how many neighbors influence the position of a given layer^54,55^, was probably S = 0 (random interstratification). This type of stacking defect has been described repeatedly for AFm during SO_4_^2−^/I^−^ exchange^8^, for C-S-H (the main cement phase)^43,56^, and more generally for several types of layered materials, including LDH and clay minerals^54,57,58^.

To provide further structural constraints on the mechanisms of Mo sorption, and to constrain better the Mo sorption sites, high-energy X-ray scattering data were converted to pair-distribution functions (PDFs). These data are therefore represented as interatomic distances in real space (Fig. 2b–d). To assign atomic pairs to the observed correlations, data from the unreacted sample were fitted using the AFm model proposed by Renaudin *et al*.^53^ (Figure S1 and Table S4). This analysis revealed that the unreacted sample was pure AFm and that the overall PDF signal was dominated by the signal from Ca-Ca and Ca-Al atomic pairs, while the low *r* part of the signal contained correlations from the first oxygen shells of Al and Ca (Al-O_1_ and Ca-O_1_, with respective distances of 1.89 Å and 2.42 Å).

**Figure 4:**
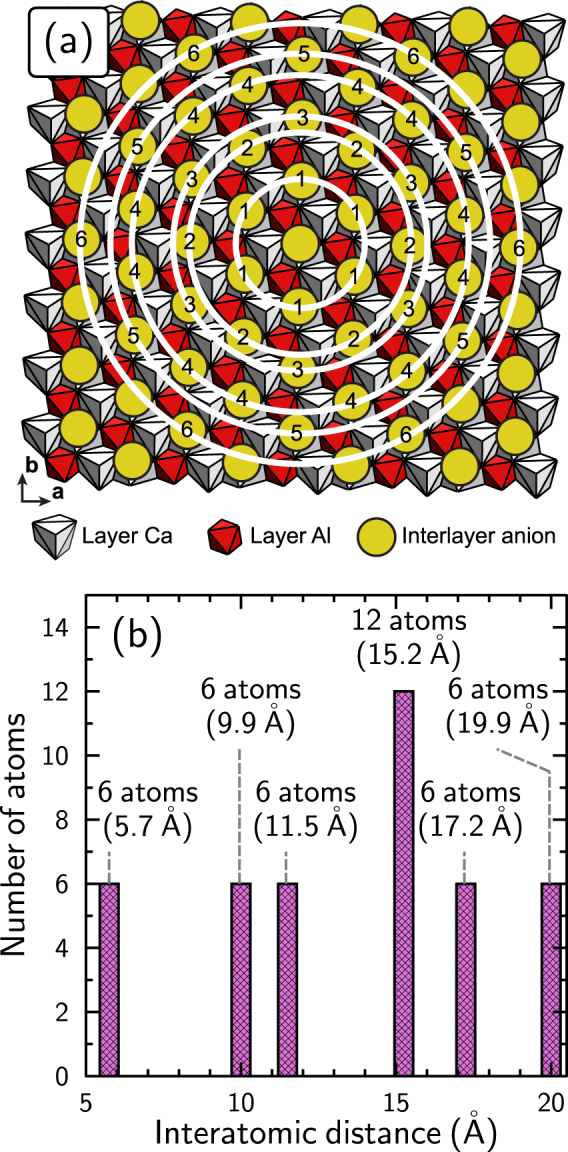
(**a**) Schematic view of AFm structure, seen perpendicular to the ab plane (layer plane). White and red polyhedra show the layered Ca and Al coordination spheres, respectively, and yellow dots are the interlayer anions (Cl or Mo). The concentric white circles highlight the presence of several shells of interlayer anions around a given interlayer anion. The shell to which a given interlayer anion belongs is noted with a number. (**b**) Number of atoms involved in each shell schematized in (**a**) as a function of the distance between the central atom and those forming the shell. Note that the interatomic distances match those increasing in intensity in Fig. 2b. Analysis arbitrarily restricted to distances *r* < 20 Å.

The PDF data of the samples that were interacted with MoO_4_^2−^ revealed several changes compared with the PDF data of the unreacted sample (Fig. 2b). The most obvious change was an increase in the intensity of the correlations attributed, in the PDF of the unreacted sample, to Cl-Cl pairs from a given interlayer (Figs 2b, 4). For example, between the PDFs of the unreacted sample and of that issued from experiment 2, the intensity of the first six of these correlations (up to ~20 Å) increased by a mean value of 85%. The study of possible changes in the intensity of the correlations involving Cl atoms from successive interlayers (along c*) was hampered by the changes in and multiplicity of the layer-to-layer distance upon Mo sorption. The increase in the intensity of the correlation attributed to Cl-Cl pairs in the unreacted sample was interpreted as an increase in the electron density at the Cl sites upon incorporation of MoO_4_^2−^. The occupancy of the Cl site in the unreacted sample was 1 and the atomic scattering factor of Mo is ~3–4 times higher than that of Cl over the diffraction angles investigated^59,60^. Therefore, such an increase was explained by the incorporation of MoO_4_^2−^ in the AFm structure that occurred through replacement of Cl^−^ at the same crystallographic position. Interestingly, the increase in intensity depended on the Mo-Mo pair considered (Mo-Mo_*x*_, where *x* is the rank of the pair, i.e., the numbers printed in Fig. 4a). More precisely, the Mo-Mo_4_ correlation (at 15.2 Å) remained of constant intensity, while for all other pairs up to Mo-Mo_6_ (at 19.9 Å), it increased in intensity (Fig. 2b). This suggests a long-range ordering of MoO_4_^2−^ in the interlayer, possibly related to the regular alternation along **b** (Fig. 4a) of “rows” preferentially filled with MoO_4_^2−^ and of “rows” depleted in MoO_4_^2−^. However, it could not be assessed quantitatively through data modeling because of the mineralogical heterogeneity of the finals solids (i.e., the presence of interlayer Cl^−^, MoO_4_^2−^, and OH^−^) and their complexity (i.e., interstratification). More generally, the fact that Mo-Mo pairs were observed at the same distance as the Cl-Cl pairs, up to the separation distance of 2 nm, is remarkable support for the hypothesis that Mo sorption by AFm proceeds through anion exchange. However, it does not give the exchange stoichiometry, which was obtained through modeling of the chemical data (see below).

A second important modification of the PDF data upon Mo exchange occurred in the Al-O_1_ correlation (Fig. 2c). The intensity of this correlation increased with an increasing degree of Mo incorporation, and the maximum of the correlation was displaced toward low *r* values. Given Al is located in the AFm layer, no significant change to its coordination environment was expected upon modification of the interlayer composition. To investigate the origin of this modification further, the PDF data from the unreacted sample were subtracted from that of the reacted samples. This provided differential PDF (d-PDF) data where it could be observed that the apparent modification of the Al-O_1_ correlation was in fact due to the presence of a peak at 1.78 Å (Fig. 2d), which increased in intensity with an increasing degree of Mo incorporation within the structure. This 1.78 Å distance is fully coherent with the presence of interlayer MoO_4_^2−^, as the same distance was observed by extended X-ray absorption fine structure spectroscopy for the Mo-O distance in tetrahedral MoO_4_^2−^ ^21^. Finally, the d-PDF data showed the presence of a broad correlation at ~3.75–4.30 Å (Fig. 2d), which was certainly because of correlations involving Mo and the closest layer of O, Al, and Ca atoms when the layer-to-layer distance was 8.60 Å.

To sum up, both the analysis of high-energy X-ray scattering and the EPMA data evidence that the degree of Mo for Cl exchange increased in the order: unreacted sample, experiment 1, experiment 3, experiment 2. Mo was incorporated in the AFm interlayer under the form of tetrahedral MoO_4_^2−^, taking the same crystallographic position as Cl^−^ in the unreacted sample, supporting the idea that Mo incorporation results mainly from an exchange reaction involving Cl^−^. All these data were then used for interpreting the chemical data collected during the flow-through experiments.

### Evolution of Mo and Cl concentration during flow-through experiments

**Figure 5:**
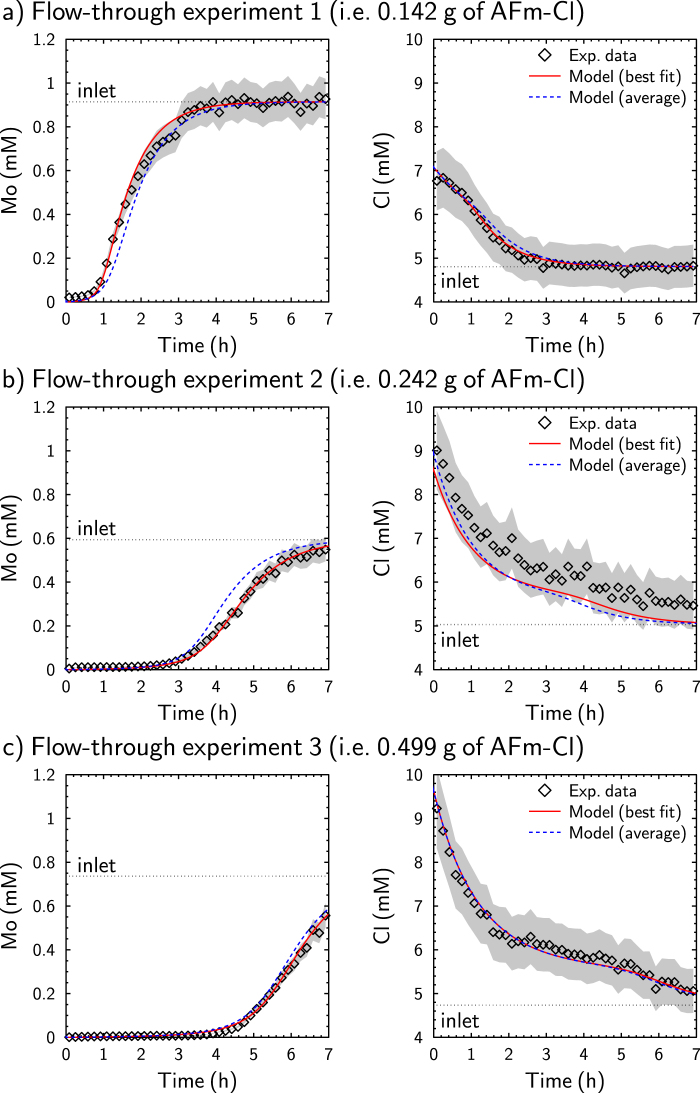
Evolutions of experimental and modeled Mo and Cl concentrations as a function of time. “Best fit” models were obtained using different AEC (Table 5), while “average” models considered the same AEC_site 1_ (230 meq. 100 g^−1^) and AEC_site 2_ (126.3 meq. 100 g^−1^) for all experiments (see text). Gray shading represents estimated error on experimental data (i.e., 10%).

As discussed above, the three flow-through experiments allowed the different stages of Mo sorption by AFm-Cl to be investigated. The evolution of Mo and Cl concentrations at the outlet of the reactor reflects these different stages (Fig. 5). In experiment 1, the release of Mo started after about 1 h of the flow-through experiment (>fluid residence time, i.e., 42 min) and a steady state could be observed, while in experiment 2, Mo was released after 3 h. In both experiments, the initial release of Cl was high. Analysis of experiment 3 provides greater insight into this high release of Cl.

Output Ca concentrations were systematically similar to the input concentrations (Supplementary Information: Figure S2). No preferential release of Ca was observed at the beginning of the flow-through experiments, confirming the absence of CaCl_2_ impurities mixed with the synthesized AFm-Cl. Output Al concentrations were scattered but they remained close to the input concentrations (except for the first hour of experiment 1, see Supplementary Information: Figure S2). Because no significant releases of Ca and Al were observed, AFm dissolution during the flow-through experiments was negligible. The pH values remained constant over time (Supplementary Information: Figure S3). Because of the continuous renewing of the reacting solutions, AFm-Cl, AFm-OH, and AFm-MoO_4_ were undersaturated (Supplementary Information: Figure S3); thus, precipitation reactions were not expected (except for two samplings of experiment 1 where SI_AFm-MoO4_ ~ 0).

### Modeling of flow-through experiments and determination of exchange parameters

#### Exchange parameters

The details of the preliminary modeling are provided in the Supplementary Information. Calculations demonstrate the importance of an integrated approach combining physical characterizations, chemical analyses (for both solutions and solids), and the consideration of all exchangeable populations (i.e., OH^−^, Cl^−^, and MoO_4_^2−^) for the determination of the selectivity constants.

As the dissolution of AFm can be neglected (see above), the amount of exchanger remained constant. The best fit of the experimental data, performed after modeling of the transport parameters (Supplementary Information: Figure S4), was obtained by considering two exchangeable sites. Cl^−^, MoO_4_^2−^, and OH^−^ were allowed to compete for adsorption on site 1, while only monovalent anions could compete for adsorption on site 2 (i.e., Cl^−^/OH^−^ exchange only). The total amount of exchanger adhered to the following relation:

2 $$AE{C}_{theoretical}=AE{C}_{site1}+AE{C}_{site2}$$

where *AEC*_*theoretical*_ is calculated from the ideal mineral formula of the AFm (i.e., 356.3 meq. 100 g^−1^ AFm or 2 eq mol^−1^ AFm), *AEC*_*site 1*_ is the AEC fitted for OH^−^/Cl^−^/MoO_4_^2−^ exchange reactions, and *AEC*_*site 2*_ is the AEC for which only the OH^−^/Cl^−^ exchange reaction occurs.

The best fit to the experimental data (Fig. 5) was obtained using identical selectivity constants in all experiments (log K_OH_ = −0.8 both for sites 1 and 2 and log K_Mo_ = 1.3 for site 1, Table 5). In contrast, various AECs were fitted: *AEC*_*site 1*_ was in the range 178–260 meq. 100 g^−1^, while *AEC*_*site 2*_ was in the range 120–178 meq. 100 g^−1^ (Table 5). Qiu *et al*.^61^ reported a collapse of the AFm interlayer in the presence of interlayer OH^−^ that inhibited the adsorption of B(OH)_4_^−^. In our experiments, the fitted *AEC*_*site 1*_ increased from experiment 1 to 3 to 2, consistent with the measured pH of the “initial reactor solution” decreasing from experiments 1 to 3 to 2 (Table 3). Nonetheless, despite these expected AEC variations, a reasonable fit of all experiments was obtained with fixed values of 230 and 126.3 meq. 100 g^−1^ for sites 1 and 2, respectively (Fig. 5).

**Table 5:** Fitted exchange parameters and AFm compositions calculated at the end of flow-through experiments. AFm compositions were established from exchanger compositions. AfmCl, AfmOH, and Afm_2_MoO_4_ correspond to species reported in Equations 3–5.

Exp. n°	AEC (meq. 100 g^−1^)		log K_Mo_	log K_OH_	Exchanger composition (equivalent fraction)			Anionic composition (mole of element per mole of AFm)		
	Site 1	Site 2				Site 1	Site 2	Mo	Cl	OH
**1**	178.1	178.2	1.3	−0.8	Afm_2_MoO_4_	0.92	—	0.5 (0.4 ± 0.3)*	0.6 (0.8 ± 0.5)*	0.5
					AfmCl	0.04	0.54			
					AfmOH	0.03	0.46			
**2**	260.3	96.0	1.3	−0.8	Afm_2_MoO_4_	0.91	—	0.7 (0.7 ± 0.1)*	0.4 (0.2 ± 0.0)*	0.3
					AfmCl	0.05	0.57			
					AfmOH	0.04	0.43			
**3**	235.8	120.5	1.3	−0.8	Afm_2_MoO_4_	0.91	—	0.6 (0.6 ± 0.1)*	0.5 (0.4 ± 0.1)*	0.3
					AfmCl	0.05	0.58			
					AfmOH	0.04	0.42			

*Measured by EPMA (Table 4).

The estimated AECs for site 1 were systematically lower than the theoretical AEC (i.e., 356.3 meq. 100 g^−1^) irrespective of the modeling assumptions and considered experiments. Therefore, monovalent anions (i.e., Cl^−^ and to a lesser extent, OH^−^) in the interlayer position were not fully exchangeable with MoO_4_^2−^. This could result from partial accessibility of exchangeable sites, as reported previously for boron^61^, and/or because of high thermodynamic stability of the interstratified AFm containing Mo and monovalent anions, as reported by Mesbah *et al*.^62^ for Kuzel’s salt. To validate the modeling procedure, the chemical composition of the solid at the end of the experiment was calculated (Table 5) and found consistent with the EPMA data (Table 4); thus, confirming the robustness of the proposed geochemical model.

#### Evidence of Cl/OH exchange reactions

Modeling of the leaching experiment was performed to validate the proposed Cl^−^/OH^−^ exchange. In these experiments, solutions were slightly undersaturated and oversaturated with respect to AFm-Cl and gibbsite, respectively (SI_AFm-Cl_ ~ −0.49 and SI_Gibbsite_ ~ 0.86, Table 2).

**Figure 6:**
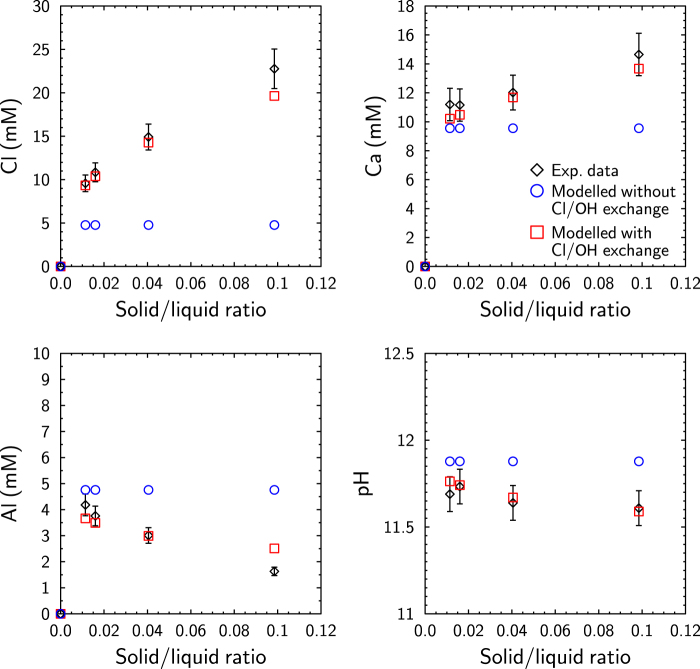
Evolution of experimental and modeled Cl, Ca, and Al concentrations and pH as a function of solid/liquid ratios (leaching experiment). An error of 10% was considered for the measured concentrations, while an error of 0.1 unit was assumed for the pH measurements.

Data were modeled in a first step assuming congruent AFm-Cl dissolution until undersaturation of −0.49, while allowing gibbsite to precipitate at an oversaturation index of 0.86. This model was unable to reproduce the experimental data (Fig. 6). In a second step, the K_OH_ selectivity constant, fitted from the flow-through experiments, was implemented and the same AEC was used. The modeled Cl concentrations then increased with the solid concentration (Fig. 6). Modeled Ca and Al concentrations, as well as the pH, were also in agreement with the experimental data over the entire range of investigated S/L ratios. More specifically, the highest amount of exchanger led to increases in both OH^−^ uptake and Cl^−^ release capacities. Therefore, the Cl^−^ concentration increased concomitantly with pH decrease, leading to the highest AFm dissolution (i.e., AFm solubility vs. pH) and explaining the Ca behavior. Finally, the lowest pH favored the stability of gibbsite (i.e., gibbsite solubility vs. pH), consistent with the observed evolution of Al concentration. Such modeling validated the proposed Cl^−^/OH^−^ exchange process.

## Conclusions

The objective of the present study was to describe quantitatively the mechanisms of Mo uptake by AFm, and to provide a geochemical model valid at both macroscopic (chemical) and molecular (crystallographic) scales. It was demonstrated that Mo, under the form of a tetrahedral MoO_4_^2−^ complex, binds to AFm by replacing 2 Cl^−^ in the interlayer mid-plane. The affinity constant was evaluated to K_Mo_ = 10^1.3^. In addition, OH^−^ competes with MoO_4_^2−^ for sorption at the same sorption site, which also prevents MoO_4_^2−^ accessing part of the AEC. Both these effects reduce the AFm sorption capacity toward Mo; thus, lowering the capacity of cement-based materials to buffer Mo.

Although this study focused on AFm, it should be remembered that this phase belongs to the LDH group of materials, which have been investigated intensively with regard to numerous applications that include depollution, industrial, and pharmacological processes. Batch and flow-through experiments, combined with geochemical data modeling and crystallographic characterization, appear powerful tools with which to investigate the exchange reactions occurring in layered materials. It is proposed that the present methodology could be generalized and extended to investigate other LDHs and/or exchangeable anions, which would enhance the understanding of the involved mechanisms and allow the determination of selectivity constants.

## Methods

### Analytical procedure

#### Solution analysis

The pH was monitored continuously (Fig. 1) using a Metrohm electrode connected to a Mettler Toledo pH meter, which was calibrated before each experiment. The solution collected at the output was divided in three aliquots. The first was used for Cl analysis using ion chromatography (Thermo-Dionex ICS3000; detection limits – dl = 0.5 mg L^−1^). The second and third aliquots were acidified using nitric acid (65% Suprapur^®^) and used respectively for determination of Ca, Na, and K concentrations using an ICP-AES (OPTIMA 5300 DV, Perkin Elmer; dl = 0.5 mg L^−1^ for all elements) and Al and Mo concentrations using an ICP-MS (NEXION 350X, Perkin Elmer; dl = 0.5 and 0.05 µg L^−1^ for Al and Mo, respectively).

#### Solid analysis

The leaching experiment was performed in an N_2_-filled glove box using various masses of synthesized AFm-Cl and ultrapure water (resistivity = 18.2 MΩ cm). The experiment lasted 10 min and the obtained solutions were then filtered using a 0.1-µm filter prior to analysis.

An electron probe microanalyzer (EPMA) was used to determine the chemical composition after the flow-through experiments (CAMECA SX FIVE). Matrix corrections were performed using a ZAF program^63^.

High-energy X-ray diffraction data were collected at station CRISTAL from SOLEIL synchrotron (Orsay, France). The energy of the incident X-rays was 28 keV (λ = 0.4367 Å). Data were collected using an XPad hybrid pixel detector in the 1.5–130° 2θ range and processed using specific software^64^ to obtain diffraction patterns. After subtraction of signal arising from the empty capillary, these patterns were processed further to produce X-ray pair-distribution function (PDF) data using PDFGetX3^65^ and a *q*-range of 0.4–17 Å^−1^. PDF data simulation was performed using PDFGui^66^ and a previously published crystal structure of AFm^53^ as a starting model. The *q* broadening and *q* dampening factors were 0.035 and 0.025 Å^−1^, respectively.

### Geochemical modeling

PHREEQC3^67^ and the THERMOCHIMIE database^68^ version 9 were used to determine the solution saturation indices and to model the flow-through and leaching experiments. The thermodynamic constant at 25 °C of the AFm-MoO_4_ was extracted from Ma *et al*.^21^.

Exchange reactions were implemented following the convention of Gaines and Thomas^69^. The exchange between the macroscopic sorption site (hereafter, AFm^+^) and Cl^−^ was assumed as a reference (arbitrary choice). Therefore, the logarithm of the selectivity constant (K_Cl_) was set to zero:

3 $$\begin{array}{c}{{\rm{Afm}}}^{+}+{{\rm{Cl}}}^{-}={\rm{AfmCl}}\\ \mathrm{log}\,{{\rm{K}}}_{{\rm{Cl}}}=0\end{array}$$

where Afm^+^ is the exchanger and Cl^−^ refers to the exchangeable anion.

In addition, the exchange reaction between OH^−^ and AFm^+^ was also taken into account:

4 $${{\rm{Afm}}}^{+}+{{\rm{OH}}}^{-}={\rm{AfmOH}}$$

Estimation of the logarithm of the selectivity constant (log K_OH_) was obtained from the fitting of experimental data.

Finally, the exchange reaction between AFm^+^ and MoO_4_^2−^ was implemented as follows:

5 $$2{{\rm{Afm}}}^{+}+{{\rm{MoO}}}_{4}^{2-}={{\rm{Afm}}}_{2}{{\rm{MoO}}}_{4}$$

The value of the selectivity constant (log K_Mo_) was also fitted using experimental data.

## Electronic supplementary material

Supplementary datafile
