# Supplementary material for: Thermodynamic and crystallographic model for anion uptake by hydrated calcium aluminate (AFm): an example of molybdenum
Source: Sci Rep. 2018 May 21;8:7943. doi: 10.1038/s41598-018-26211-z (PMC5962639; doi:10.1038/s41598-018-26211-z)
Supplement: Supplementary file 1 — Supplementary datafile [file 41598_2018_26211_MOESM1_ESM.docx]

**Supplementary Information**

**Thermodynamic and crystallographic model for anion uptake by hydrated calcium aluminate (AFm): an example of molybdenum**

Nicolas C.M. Marty^1,*,+^, Sylvain Grangeon^1,+^, Erik Elkaïm^2^, Christophe Tournassat^1,3,4^, Clémence Fauchet^1^, Francis Claret^1^

^1^ BRGM, 3 Avenue Guillemin, Orléans Cedex 2, 45060, France

^2^ Synchrotron SOLEIL, L’Orme des Merisiers Saint-Aubin, BP 48, 91192 Gif-sur-Yvette Cedex, France

^3^ ISTO UMR 7327 Université d’Orléans–CNRS– BRGM, 45071 Orléans, France

^4^ Lawrence Berkeley National Laboratory, 1 Cyclotron Road, Mail Stop 90-1116, Berkeley, CA 94720, United States

^+^ These authors contributed equally to this work

* Corresponding author: n.marty@brgm.fr

| **Sample** | **Flow rate (mL min^−1^)** | **Time**  **(h)** | **pH** | **Mo**  **(mM)** | **Cl**  **(mM)** | **Ca**  **(mM)** | **Na**  **(mM)** | **K**  **(mM)** | **Al**  **(µM)** |
| --- | --- | --- | --- | --- | --- | --- | --- | --- | --- |
| 1 | 2.01 | 0.08 | 12.16 | 0.021 | 6.77 | 13. 8 | 0.35 | 4.95 | 125 |
| 2 | 2.03 | 0.25 | 12.24 | 0.023 | 6.84 |  |  |  |  |
| 3 | 2.02 | 0.42 | 12.30 | 0.026 | 6.72 | 14.2 | 2.23 | 3.05 | 185 |
| 4 | 2.03 | 0.58 | 12.34 | 0.032 | 6.58 |  |  |  |  |
| 5 | 2.03 | 0.75 | 12.35 | 0.049 | 6.50 | 14.2 | 3.39 | 1.83 | 149 |
| 6 | 2.02 | 0.92 | 12.35 | 0.092 | 6.32 |  |  |  |  |
| 7 | 2.02 | 1.08 | 12.36 | 0.176 | 6.08 | 14.2 | 4.14 | 1.10 | 113 |
| 8 | 2.02 | 1.25 | 12.35 | 0.287 | 5.87 |  |  |  |  |
| 9 | 2.03 | 1.42 | 12.34 | 0.364 | 5.69 | 14.0 | 4.53 | 0.66 | 87.2 |
| 10 | 2.02 | 1.58 | 12.34 | 0.447 | 5.47 |  |  |  |  |
| 11 | 2.02 | 1.75 | 12.33 | 0.512 | 5.40 | 14.1 | 4.80 | 0.41 | 57.3 |
| 12 | 2.02 | 1.92 | 12.33 | 0.574 | 5.22 |  |  |  |  |
| 13 | 2.02 | 2.08 | 12.32 | 0.630 | 5.18 | 13.9 | 5.01 | 0.34 | 46.6 |
| 14 | 2.01 | 2.25 | 12.32 | 0.669 | 5.06 |  |  |  |  |
| 15 | 2.02 | 2.42 | 12.32 | 0.711 | 4.96 | 13.9 | 4.98 | 0.16 | 42.4 |
| 16 | 2.03 | 2.58 | 12.31 | 0.732 | 5.00 |  |  |  |  |
| 17 | 2.02 | 2.75 | 12.31 | 0.748 | 4.98 | 14.0 | 5.12 | 0.10 | 38.9 |
| 18 | 2.03 | 2.92 | 12.31 | 0.760 | 4.77 |  |  |  |  |
| 19 | 2.01 | 3.08 | 12.30 | 0.831 | 4.89 | 14.1 | 5.21 | 0.07 | 38.9 |
| 20 | 2.02 | 3.25 | 12.30 | 0.867 | 4.87 |  |  |  |  |
| 21 | 2.02 | 3.42 | 12.30 | 0.877 | 4.85 | 14.2 | 5.28 | 0.05 | 33.8 |
| 22 | 2.02 | 3.58 | 12.29 | 0.896 | 4.83 |  |  |  |  |
| 23 | 2.02 | 3.75 | 12.29 | 0.885 | 4.82 | 14.0 | 5.21 | 0.04 | 33.5 |
| 24 | 2.03 | 3.92 | 12.28 | 0.913 | 4.84 |  |  |  |  |
| 25 | 2.03 | 4.08 | 12.28 | 0.866 | 4.83 | 14.1 | 5.23 | 0.03 | 31.4 |
| 26 | 2.03 | 4.25 | 12.27 | 0.912 | 4.85 |  |  |  |  |
| 27 | 2.02 | 4.42 | 12.27 | 0.923 | 4.85 | 14.1 | 5.25 | 0.03 | 31.9 |
| 28 | 2.02 | 4.58 | 12.26 | 0.904 | 4.83 |  |  |  |  |
| 29 | 2.02 | 4.75 | 12.26 | 0.932 | 4.78 | 14.1 | 5.26 | 0.03 | 27.6 |
| 30 | 2.02 | 4.92 | 12.25 | 0.913 | 4.81 |  |  |  |  |
| 31 | 2.02 | 5.08 | 12.24 | 0.908 | 4.65 | 14.0 | 5.21 | 0.02 | 28.9 |
| 32 | 2.02 | 5.25 | 12.24 | 0.886 | 4.76 |  |  |  |  |
| 33 | 2.03 | 5.42 | 12.23 | 0.910 | 4.79 | 14.2 | 5.24 | 0.03 | 28.7 |
| 34 | 2.03 | 5.58 | 12.24 | 0.918 | 4.81 |  |  |  |  |
| 35 | 2.02 | 5.75 | 12.24 | 0.915 | 4.83 | 14.0 | 5.12 | 0.02 | 29.1 |
| 36 | 2.02 | 5.92 | 12.22 | 0.938 | 4.82 |  |  |  |  |
| 37 | 2.02 | 6.08 | 12.22 | 0.907 | 4.78 | 14.1 | 5.18 | 0.02 | 28.2 |
| 38 | 2.02 | 6.25 | 12.22 | 0.868 | 4.74 |  |  |  |  |
| 39 | 2.02 | 6.42 | 12.23 | 0.906 | 4.80 | 14.1 | 5.21 | 0.02 | 28.4 |
| 40 | 2.02 | 6.58 | 12.22 | 0.896 | 4.80 |  |  |  |  |
| 41 | 2.02 | 6.75 | 12.22 | 0.937 | 4.81 | 14.1 | 5.27 | 0.02 | 28.6 |
| 42 | 2.02 | 6.92 | 12.22 | 0.927 | 4.83 |  |  |  |  |

**Table S1.** Monitored flow rates, output pH, and concentrations from flow-through experiment 1 (i.e., using 0.142 g of AFm-Cl).

| **Sample** | **Flow rate (mL min^−1^)** | **Time**  **(h)** | **pH** | **Mo**  **(mM)** | **Cl**  **(mM)** | **Ca**  **(mM)** | **Na**  **(mM)** | **K**  **(mM)** | **Al**  **(µM)** |
| --- | --- | --- | --- | --- | --- | --- | --- | --- | --- |
| 1 | 2.00 | 0.08 | 12.25 | 0.005 | 9.01 | 13. 6 | 0.37 | 5.28 | 6.0 |
| 2 | 1.99 | 0.25 | 12.27 | 0.011 | 8.70 |  |  |  |  |
| 3 | 1.99 | 0.42 | 12.30 | 0.011 | 8.38 | 13.4 | 2.20 | 3.16 | 25.8 |
| 4 | 2.00 | 0.58 | 12.30 | 0.011 | 7.93 |  |  |  |  |
| 5 | 2.00 | 0.75 | 12.30 | 0.011 | 7.68 | 13.5 | 3.37 | 1.91 | 12.7 |
| 6 | 2.00 | 0.92 | 12.30 | 0.012 | 7.53 |  |  |  |  |
| 7 | 2.00 | 1.08 | 12.29 | 0.012 | 7.24 | 13.4 | 4.09 | 1.14 | 19.8 |
| 8 | 2.00 | 1.25 | 12.29 | 0.012 | 7.02 |  |  |  |  |
| 9 | 1.99 | 1.42 | 12.28 | 0.012 | 7.12 | 12.2 | 4.51 | 0.70 | 92.7 |
| 10 | 2.00 | 1.58 | 12.28 | 0.013 | 6.83 |  |  |  |  |
| 11 | 1.99 | 1.75 | 12.27 | 0.013 | 6.68 | 12.7 | 4.75 | 0.43 | 76.2 |
| 12 | 1.99 | 1.92 | 12.26 | 0.015 | 6.71 |  |  |  |  |
| 13 | 1.99 | 2.08 | 12.26 | 0.015 | 7.00 | 12.8 | 4.91 | 0.27 | 107 |
| 14 | 1.99 | 2.25 | 12.25 | 0.017 | 6.54 |  |  |  |  |
| 15 | 2.00 | 2.42 | 12.24 | 0.020 | 6.39 | 12.9 | 5.00 | 0.16 | 49.9 |
| 16 | 2.00 | 2.58 | 12.24 | 0.023 | 6.26 |  |  |  |  |
| 17 | 1.99 | 2.75 | 12.24 | 0.029 | 6.32 | 12.2 | 4.99 | 0.11 | 72.2 |
| 18 | 1.99 | 2.92 | 12.24 | 0.038 | 6.35 |  |  |  |  |
| 19 | 1.99 | 3.08 | 12.23 | 0.049 | 6.06 | 11.6 | 5.12 | 0.07 | 40.5 |
| 20 | 1.99 | 3.25 | 12.23 | 0.063 | 6.18 |  |  |  |  |
| 21 | 1.98 | 3.42 | 12.23 | 0.080 | 6.03 | 12.1 | 5.07 | 0.05 | 71.0 |
| 22 | 1.99 | 3.58 | 12.22 | 0.105 | 6.36 |  |  |  |  |
| 23 | 1.99 | 3.75 | 12.22 | 0.133 | 6.15 | 12.2 | 4.87 | 0.04 | 37.3 |
| 24 | 1.98 | 3.92 | 12.21 | 0.156 | 6.14 |  |  |  |  |
| 25 | 1.99 | 4.08 | 12.19 | 0.195 | 6.36 | 12.2 | 4.73 | 0.03 | 25.8 |
| 26 | 1.98 | 4.25 | 12.19 | 0.207 | 5.87 |  |  |  |  |
| 27 | 1.98 | 4.42 | 12.20 | 0.260 | 5.84 | 12.6 | 4.82 | 0.03 | 22.0 |
| 28 | 1.99 | 4.58 | 12.20 | 0.259 | 5.88 |  |  |  |  |
| 29 | 1.98 | 4.75 | 12.20 | 0.325 | 5.64 | 12.2 | 4.65 | 0.02 | 111 |
| 30 | 1.99 | 4.92 | 12.20 | 0.357 | 5.88 |  |  |  |  |
| 31 | 1.98 | 5.08 | 12.21 | 0.405 | 5.64 | 11.0 | 4.80 | 0.02 | 92.4 |
| 32 | 1.98 | 5.25 | 12.21 | 0.414 | 5.83 |  |  |  |  |
| 33 | 1.98 | 5.42 | 12.22 | 0.453 | 5.60 | 12.4 | 4.90 | 0.02 | 46.3 |
| 34 | 1.97 | 5.58 | 12.22 | 0.440 | 5.45 |  |  |  |  |
| 35 | 1.98 | 5.75 | 12.23 | 0.499 | 5.75 | 11.2 | 4.87 | 0.02 | 57.7 |
| 36 | 1.97 | 5.92 | 12.23 | 0.491 | 5.57 |  |  |  |  |
| 37 | 1.97 | 6.08 | 12.24 | 0.525 | 5.53 | 11.9 | 4.79 | 0.02 | 55.2 |
| 38 | 1.97 | 6.25 | 12.24 | 0.511 | 5.55 |  |  |  |  |
| 39 | 1.96 | 6.42 | 12.25 | 0.515 | 5.48 | 12.5 | 4.78 | 0.02 | 65.7 |
| 40 | 1.96 | 6.58 | 12.25 | 0.543 | 5.60 |  |  |  |  |
| 41 | 1.97 | 6.75 | 12.24 | 0.537 | 5.48 | 13.4 | 4.84 | 0.02 | 22.7 |
| 42 | 1.97 | 6.92 | 12.26 | 0.549 | 5.46 |  |  |  |  |

**Table S2.** Monitored flow rates, output pH, and concentrations from flow-through experiment 2 (i.e., using 0.242 g of AFm-Cl).

| **Sample** | **Flow rate (mL min^−1^)** | **Time**  **(h)** | **pH** | **Mo**  **(mM)** | **Cl**  **(mM)** | **Ca**  **(mM)** | **Na**  **(mM)** | **K**  **(mM)** | **Al**  **(µM)** |
| --- | --- | --- | --- | --- | --- | --- | --- | --- | --- |
| 1 | 2.17 | 0.08 | 12.26 | 0.001 | 9.23 |  |  |  |  |
| 2 | 2.21 | 0.25 | 12.27 | 0.002 | 8.72 | 12.9 | 1.40 | 3.65 | 12.1 |
| 3 | 2.23 | 0.42 | 12.28 | 0.003 | 8.23 |  |  |  |  |
| 4 | 2.23 | 0.58 | 12.28 | 0.003 | 7.71 | 12.8 | 2.98 | 2.07 | 12.5 |
| 5 | 2.23 | 0.75 | 12.29 | 0.004 | 7.57 |  |  |  |  |
| 6 | 2.23 | 0.92 | 12.28 | 0.004 | 7.30 | 12.7 | 3.89 | 1.17 | 11.7 |
| 7 | 2.22 | 1.08 | 12.29 | 0.005 | 7.07 |  |  |  |  |
| 8 | 2.22 | 1.25 | 12.29 | 0.005 | 6.83 | 12.5 | 4.38 | 0.66 | 10.8 |
| 9 | 2.22 | 1.42 | 12.29 | 0.005 | 6.80 |  |  |  |  |
| 10 | 2.21 | 1.58 | 12.29 | 0.006 | 6.40 | 12.7 | 4.69 | 0.39 | 11.2 |
| 11 | 2.23 | 1.75 | 12.29 | 0.006 | 6.35 |  |  |  |  |
| 12 | 2.20 | 1.92 | 12.29 | 0.006 | 6.34 | 12.1 | 4.82 | 0.22 | 14.5 |
| 13 | 2.22 | 2.08 | 12.29 | 0.006 | 6.14 |  |  |  |  |
| 14 | 2.22 | 2.25 | 12.29 | 0.007 | 6.19 | 12.4 | 4.83 | 0.13 | 15.8 |
| 15 | 2.22 | 2.42 | 12.29 | 0.008 | 6.17 |  |  |  |  |
| 16 | 2.21 | 2.58 | 12.29 | 0.007 | 6.30 | 11.8 | 4.90 | 0.07 | 21.9 |
| 17 | 2.22 | 2.75 | 12.29 | 0.007 | 6.13 |  |  |  |  |
| 18 | 2.21 | 2.92 | 12.28 | 0.007 | 6.12 | 12.3 | 4.86 | 0.05 | 15.9 |
| 19 | 2.21 | 3.08 | 12.29 | 0.008 | 6.11 |  |  |  |  |
| 20 | 2.20 | 3.25 | 12.29 | 0.008 | 6.00 | 11.2 | 4.75 | 0.03 | 32.5 |
| 21 | 2.21 | 3.42 | 12.29 | 0.010 | 6.00 |  |  |  |  |
| 22 | 2.20 | 3.58 | 12.29 | 0.011 | 5.91 | 12.0 | 4.86 | 0.03 | 18.8 |
| 23 | 2.20 | 3.75 | 12.30 | 0.013 | 5.90 |  |  |  |  |
| 24 | 2.20 | 3.92 | 12.30 | 0.018 | 5.89 | 12.5 | 4.88 | 0.02 | 15.4 |
| 25 | 2.21 | 4.08 | 12.29 | 0.025 | 5.79 |  |  |  |  |
| 26 | 2.20 | 4.25 | 12.29 | 0.030 | 5.82 | 12.5 | 4.90 | 0.02 | 15.1 |
| 27 | 2.21 | 4.42 | 12.29 | 0.038 | 5.88 |  |  |  |  |
| 28 | 2.19 | 4.58 | 12.30 | 0.048 | 5.80 | 12.1 | 4.83 | 0.02 | 21.8 |
| 29 | 2.20 | 4.75 | 12.30 | 0.069 | 5.76 |  |  |  |  |
| 30 | 2.20 | 4.92 | 12.29 | 0.100 | 5.55 | 12.7 | 5.00 | 0.02 | 13.5 |
| 31 | 2.18 | 5.08 | 12.29 | 0.130 | 5.69 |  |  |  |  |
| 32 | 2.20 | 5.25 | 12.29 | 0.159 | 5.68 | 12.5 | 5.00 | 0.01 | 17.4 |
| 33 | 2.20 | 5.42 | 12.29 | 0.194 | 5.55 |  |  |  |  |
| 34 | 2.19 | 5.58 | 12.29 | 0.226 | 5.42 | 11.7 | 5.01 | 0.01 | 34.3 |
| 35 | 2.20 | 5.75 | 12.28 | 0.272 | 5.42 |  |  |  |  |
| 36 | 2.19 | 5.92 | 12.28 | 0.308 | 5.11 | 11.9 | 4.97 | 0.01 | 33.5 |
| 37 | 2.17 | 6.08 | 12.28 | 0.337 | 5.27 |  |  |  |  |
| 38 | 2.17 | 6.25 | 12.28 | 0.386 | 5.26 | 11.1 | 4.90 | 0.01 | 28.9 |
| 39 | 2.17 | 6.42 | 12.27 | 0.409 | 5.26 |  |  |  |  |
| 40 | 2.16 | 6.58 | 12.28 | 0.490 | 5.09 | 12.0 | 4.89 | 0.01 | 17.9 |
| 41 | 2.16 | 6.75 | 12.27 | 0.477 | 5.06 |  |  |  |  |
| 42 | 2.16 | 6.92 | 12.27 | 0.557 | 5.06 | 12.3 | 4.95 | 0.01 | 9.0 |

**Table S3.** Monitored flow rates, output pH, and concentrations from flow-through experiment 3 (i.e., using 0.499 g of AFm-Cl).


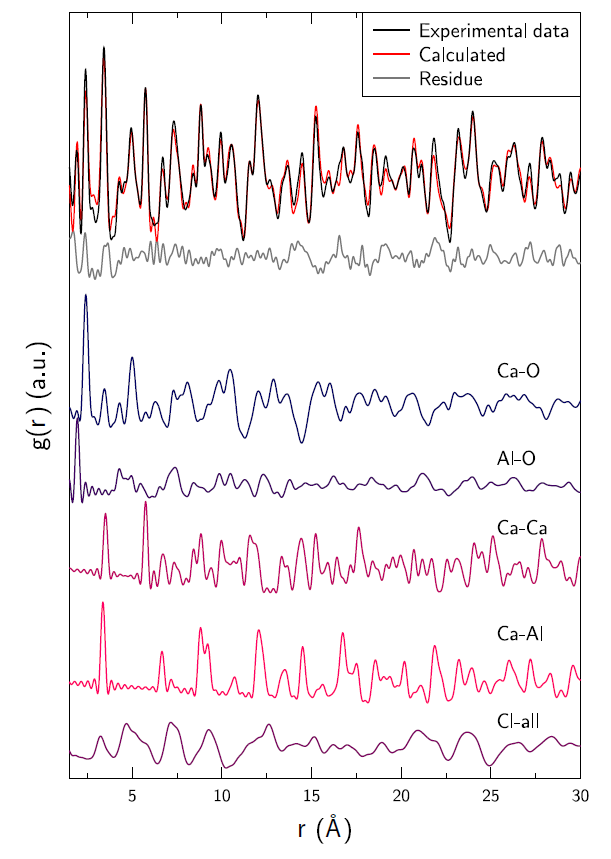


Figure S1. Top: Experimental data of the unreacted AFm (black solid line) overlaid above the calculated pattern (red solid line) and residual (grey solid line). The five patterns below the residual represent the calculated partial PDFs (from top to bottom): Ca and O atoms, Al and O atoms, only Ca atoms, Ca and Al atoms, and Cl atoms. The structure model is provided in Table S4.

|  |  |  |  |  |  |  |
| --- | --- | --- | --- | --- | --- | --- |
| Atom | *x*/*a* | *y*/*b* | *z*/*c* | U_11_ (Å^2^) | U_22_ (Å^2^) | U_33_ (Å^2^) |
| Al | 3/4 | 3/4 | 0 | **0.004 ± 0.006^a^** | **0.004 ± 0.006^a^** | **0.011 ± 0.01^b^** |
| Ca | **0.0965 ± 0.0067** | **0.7546 ± 0.011** | **0.0372 ± 0.0027** | **0.004 ± 0.006^a^** | **0.012 ± 0.007** | **0.011 ± 0.01^b^** |
| O | 0.878 | 0.6513 | 0.9342 | **0.030 ± 0.056^c^** | **0.006 ± 0.026^d^** | **0.038 ± 0.05^e^** |
| O | 0.8086 | 0.47 | 0.0661 | **0.030 ± 0.056^c^** | **0.006 ± 0.026^d^** | **0.038 ± 0.05^e^** |
| O | 0.597 | 0.566 | 0.9316 | **0.030 ± 0.056^c^** | **0.006 ± 0.026^d^** | **0.038 ± 0.05^e^** |
| O | 0.1729 | 0.7453 | 0.1993 | **0.021 ± 0.044^f^** | **0.057 ± 0.087^g^** | **0.055 ± 0.068^h^** |
| Cl | 0 | 0.3283 | 0.25 | **0.021 ± 0.044^f^** | **0.057 ± 0.087^g^** | **0.055 ± 0.068^h^** |
|  |  |  |  |  |  |  |
| Space group: C2/m, ***b* = (5.7354 ± 0.0088) Å**, ***c* = (15.9834 ± 0.096)** **Å.** The ratio *a*/*b* was constrained to be equal to that of the original AFm model. Consequently, *a* = (9.9628 ± 0.015) Å.  *α* = 90°, ***β* = (102.72 ± 0.069)°**, *γ* = 90°. δ_2_ (quadratic atomic correlation factor) = 3.52, Q_broadening_ = 0.035, Q_dampening_ = 0.025, R_W_ = 27.5%. | | | | | | |
| Note: Parameters in bold are those refined as compared to the original AFm model from Renaudin and coworkers. Hydrogen atoms reported in this previous study were not included in the present simulations. Superscripts ^a^, ^b^, ^c^, ^d^, ^e^, ^f^, ^g^, and ^h^ denote parameters constrained to be equal during the refinement procedure. | | | | | | |

**Table S4.** Structure model used to reproduce the PDF data from the unreacted AFm (Figure S1).


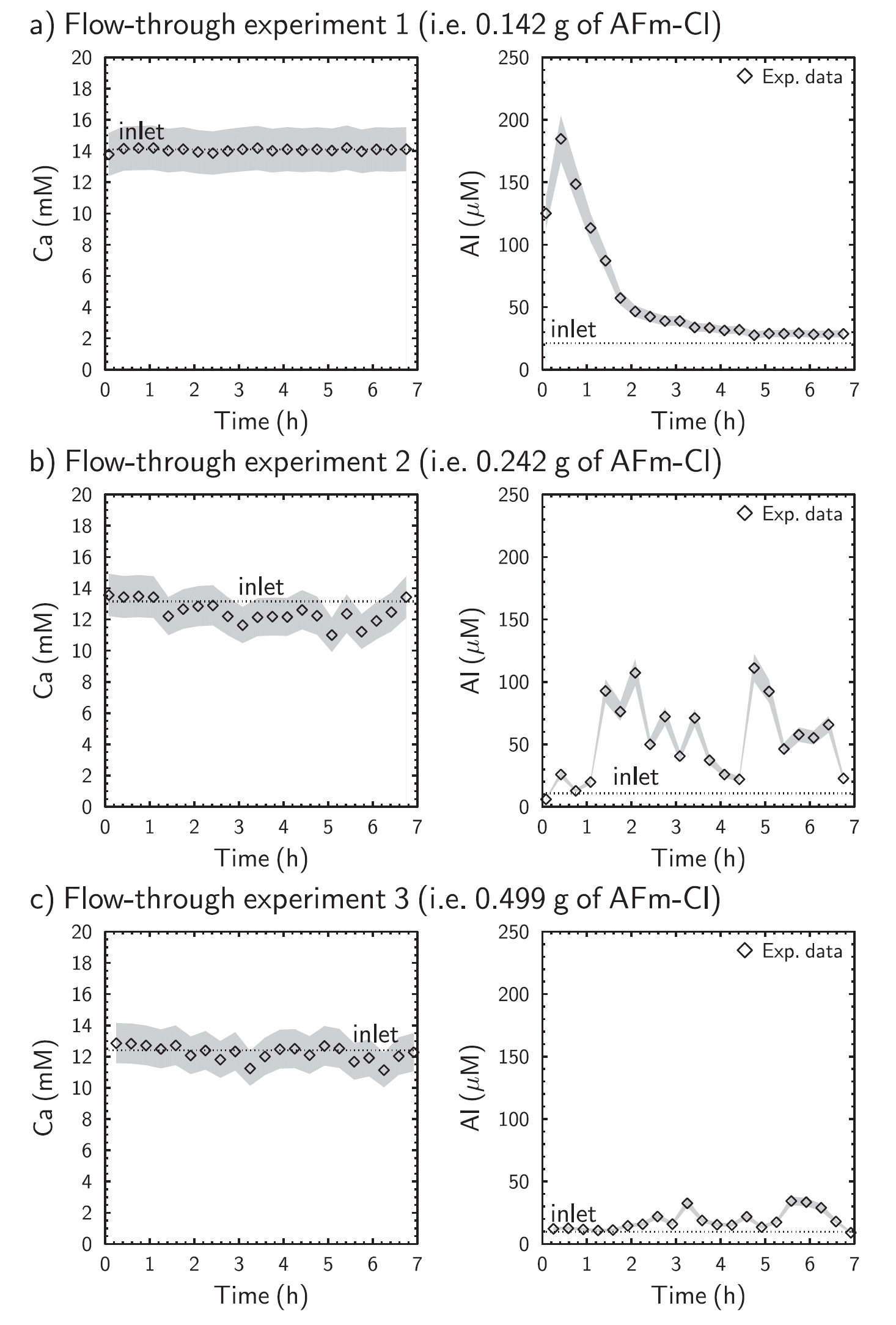


Figure S2. Evolutions of experimental Ca and Al concentrations as a function of time. Gray shading represents estimated error on experimental data (i.e., 10%).


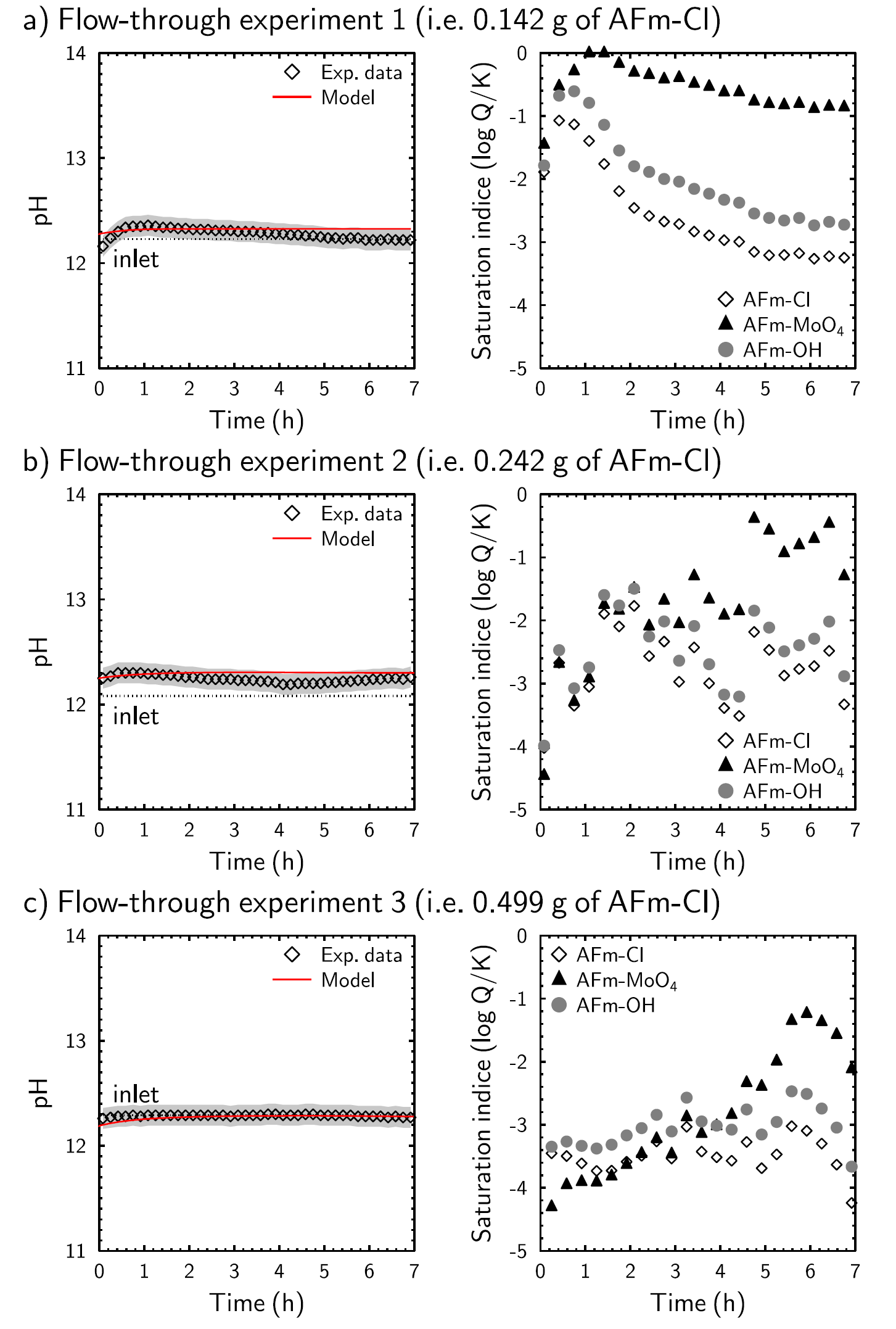


Figure S3. Evolutions of experimental and modeled pH, as well as saturation indices calculated from solution chemistries, as a function of time (AFm-Cl, AFm-OH, and AFm-MoO_4_). Gray shading represents estimated error on pH measurements (i.e., 0.1 unit).


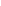

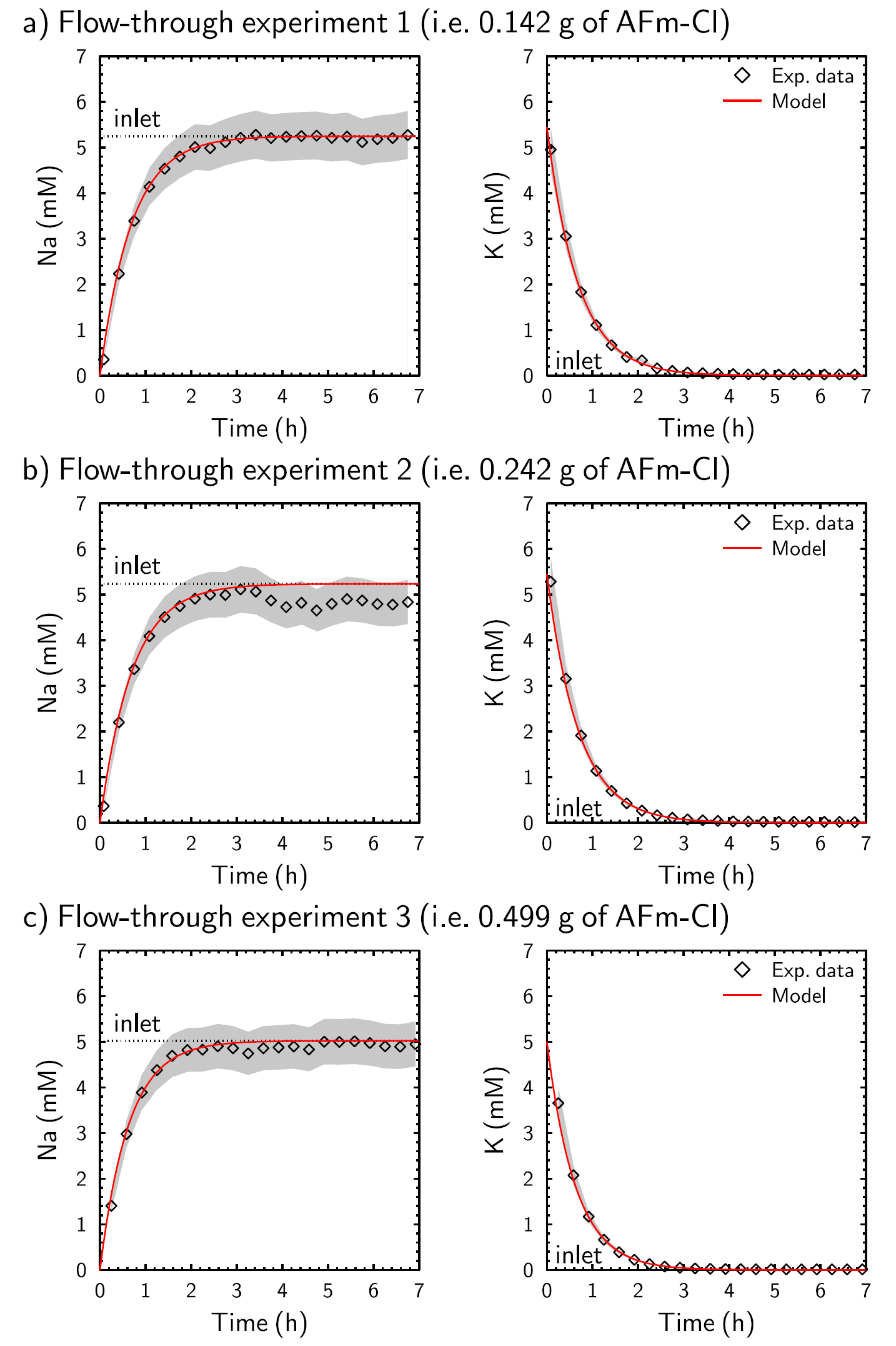


Figure S4. Evolutions of experimental and modeled Na and K concentrations as a function of time. Gray shading represents estimated error on experimental data (i.e., 10%).

Preliminary Modeling

A first simulation (model 1) was performed considering Cl^-^/MoO_4_^2-^ exchange as the sole possible reaction (i.e., without Cl^-^/OH^-^ exchange) and a single exchangeable site. Modeled Mo and Cl concentrations over time are presented in Figure S5. The Mo behavior was simulated correctly for the three experiments using similar values for the logarithm of the Cl^-^/MoO_4_^2-^ selectivity constant (Table S5). Nonetheless, the fitted AECs (Table S5) were lower than the theoretical values calculated from the ideal mineral formula of the AFm (i.e., 356.3 meq 100 g^−1^ or 2 eq mol^−1^). Therefore, the Cl in the interlayered position of the AFm-Cl was not fully exchangeable. Final anionic compositions were then calculated considering both the exchanger compositions and the immobile fraction of Cl (Table S5). The Mo contents were in agreement with the EPMA data, while the atomic abundances of Cl were inconsistent with chemical measurements (Table 4). Moreover, the model was unable to reproduce the high release of Cl during the first hours of the experiments (Figure S5).

| **Exp. n°** | **AEC**  **(meq 100 g^−1^)** | **log K**_Mo_ | **Exchanger composition**  **(equivalent fraction)** | | **Anionic composition**  **(mole of element per mole of AFm)** | |
| --- | --- | --- | --- | --- | --- | --- |
|  |  |  |  |  | **Mo** | **Cl** |
| **1** | 178.1 | 0.8 | Afm_2_MoO_4_ | 0.93 | 0.5  (0.4 ± 0.3)* | 1.1  (0.8 ± 0.5)* |
|  |  |  | AfmCl | 0.07 |  |  |
| **2** | 260.3 | 0.8 | Afm_2_MoO_4_ | 0.90 | 0.7  (0.7 ± 0.1)* | 0.7  (0.2 ± 0.0)* |
|  |  |  | AfmCl | 0.10 |  |  |
| **3** | 235.8 | 1.0 | Afm_2_MoO_4_ | 0.92 | 0.6  (0.6 ± 0.1)* | 0.8  (0.4 ± 0.1)* |
|  |  |  | AfmCl | 0.08 |  |  |

** Measured by EPMA (Table 4)*

**Table S5.** Final exchanger and anionic compositions calculated from model 1 (i.e., without Cl^-^/OH^-^ exchange). AfmCl and Afm_2_MoO_4,_ correspond to species reported in equations 3 and 5. AFm compositions were established from exchanger compositions and fitted AECs (see text).

A second model (model 2) was then built considering all exchangeable populations (i.e., OH^-^, Cl^-^, and MoO_4_^2-^). Mo and Cl behaviors were simulated correctly (Figure S5). Nonetheless, Cl contents calculated by summing the exchanger compositions and nonexchangeable fractions of AFm-Cl at the end of flow-through experiments (Table S6) were not in agreement with the EPMA data (Table 4).

| **Exp. n°** | **AEC**  **(meq 100 g^−1^)** | **log K**_Mo_ | **log K**_Cl_ | **Exchanger composition**  **(equivalent fraction)** | | **Anionic composition**  **(mole of element per mole of AFm)** | | |
| --- | --- | --- | --- | --- | --- | --- | --- | --- |
|  |  |  |  |  |  | **Mo** | **Cl** | **OH** |
| **1** | 178.1 | 1.6 | −0.4 | Afm_2_MoO_4_ | 0.91 | 0.5  (0.4 ± 0.3)* | 1.0  (0.8 ± 0.5)* | 0.1 |
|  |  |  |  | AfmCl | 0.03 |  |  |  |
|  |  |  |  | AfmOH | 0.06 |  |  |  |
| **2** | 260.3 | 1.6 | −0.6 | Afm_2_MoO_4_ | 0.89 | 0.7  (0.7 ± 0.1)* | 0.6  (0.2 ± 0.0)* | 0.1 |
|  |  |  |  | AfmCl | 0.04 |  |  |  |
|  |  |  |  | AfmOH | 0.07 |  |  |  |
| **3** | 235.8 | 1.6 | −0.6 | Afm_2_MoO_4_ | 0.92 | 0.6  (0.6 ± 0.1)* | 0.7  (0.4 ± 0.1)* | 0.1 |
|  |  |  |  | AfmCl | 0.04 |  |  |  |
|  |  |  |  | AfmOH | 0.04 |  |  |  |

** Measured by EPMA (Table 4)*

**Table S6.** Final exchanger and anionic compositions calculated from model 2 (i.e., with Cl^-^/OH^-^ exchange). AfmCl and Afm_2_MoO_4,_ correspond to species reported in equations 3 and 5. AFm compositions were established from exchanger compositions and fitted AECs (see text).

Finally, the best fit of the experimental data was obtained with a third model that considered all exchangeable populations (i.e., OH^-^, Cl^-^, and MoO_4_^2-^) and two exchangeable sites, as reported in the manuscript. The capabilities of the models to reproduce the experimental data are summarized in Table S7.

| **Model** | **Modeling assumption** | **Solution chemistry** | | **Solid chemistry** |
| --- | --- | --- | --- | --- |
|  |  | **Mo** | **Cl** |  |
| 1 | 1 site and Cl^-^/MoO_4_^-2^ exchange | **√** | X | X |
| 2 | 1 site and OH^-^**/**Cl^-^/MoO_4_^-2^ exchange | **√** | **√** | X |
| 3 (see manuscript) | 2 sites and OH^-^**/**Cl^-^/MoO_4_^-2^ exchange | **√** | **√** | **√** |

**Table S7.** Capabilities of models to reproduce the experimental data. For detailed descriptions of the models, see above.


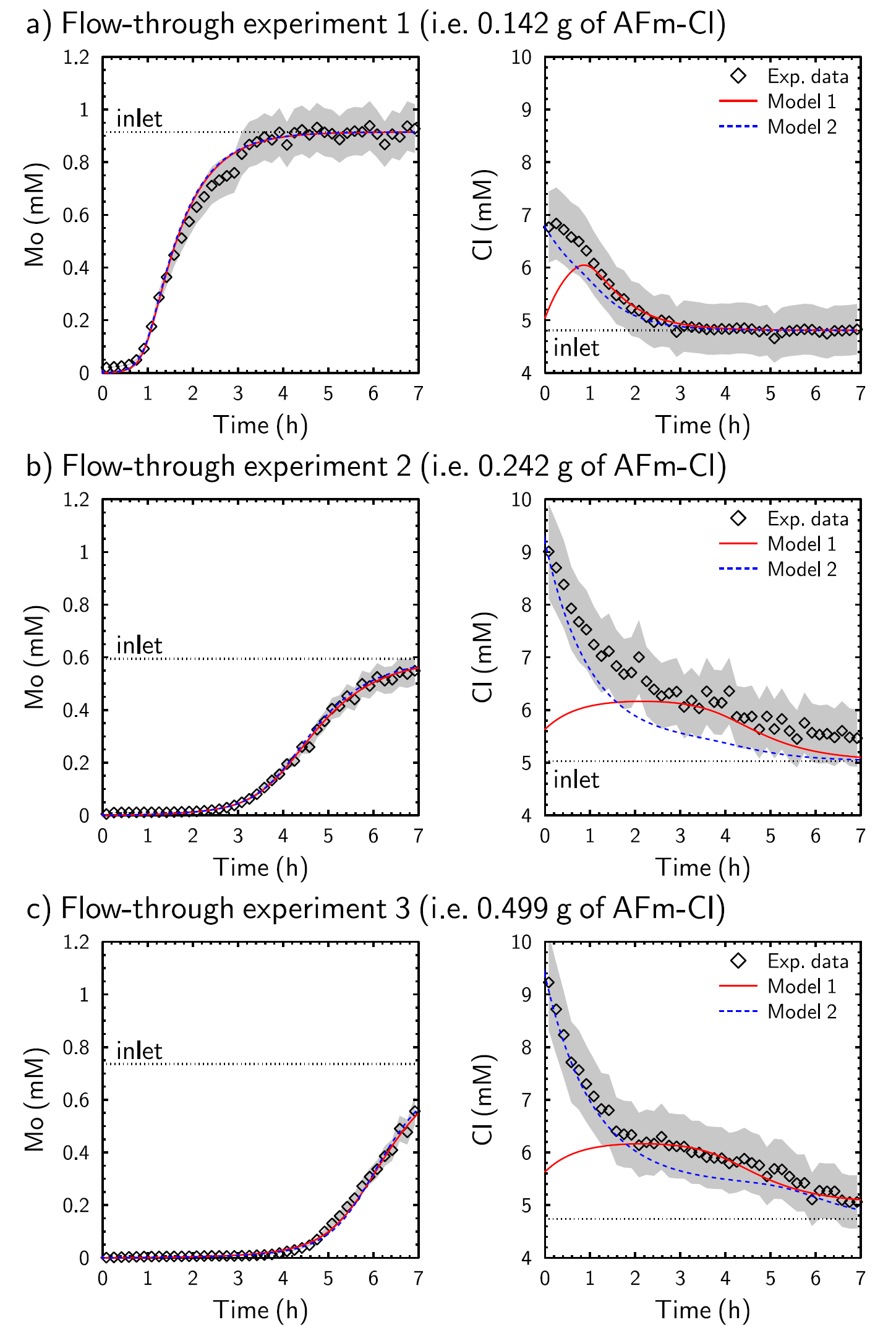


Figure S5. Evolutions of experimental and modeled Mo and Cl concentrations as a function of time (models 1 & 2, see text). Gray shading represents estimated error on experimental data (i.e., 10%).
